# A Flawed Conjecture Keeps Haunting Brain Energy Metabolism Research

**DOI:** 10.3390/neurosci7030049

**Published:** 2026-04-22

**Authors:** Avital Schurr

**Affiliations:** Department of Anesthesiology and Perioperative Medicine, School of Medicine, University of Louisville, Louisville, KY 40202, USA; avital.schurr@gmail.com

**Keywords:** active brain, energy metabolism, glucose, glycolysis, lactate, oxidative phosphorylation, pyruvate, TCA cycle

## Abstract

In 1988, two seminal studies were published almost simultaneously in the same scientific journal. Both spurred the field of brain energy metabolism research in new directions, culminating in a long-lasting debate that appeared to split its practitioners into two factions that seem unwilling to agree on what metabolic processes are fueling the active brain with adenosine triphosphate (ATP). The first study used rat hippocampal slices to demonstrate the ability of lactate to support neuronal function as the sole oxidative mitochondrial substrate. The second study demonstrated that upon brain stimulation, glucose consumption is not accompanied by respective oxygen consumption, but a non-oxidative glucose utilization or what has become known as “aerobic glycolysis”. Consequently, for almost four decades, researchers in this field have been divided between those who profess that brain activity is supported by oxidative lactate metabolism and those who insist that non-oxidative glucose metabolism supports it. Hypotheses for both concepts were offered, “The Astrocyte Neuron Lactate Shuttle Hypothesis” and “The Efficiency Tradeoff Hypothesis,” respectively. To bridge the gap between the two groups, a recent editorial, authored by over twenty leading investigators, was published. The editorial received two separate responses from investigators who supported the non-oxidative glucose consumption as the main process supporting neural activity, signaling that the gap between the two groups remained. The present perspective highlights the principal disagreements that divide this utmost important field of research. It argues that the main reason for these disagreements is rooted in the assumption that pyruvate is the end-product of aerobic glycolysis, even when many among those who adhere to this assumption accept that in the active brain glycolysis is the main provider of the necessary ATP and the end-product is lactate under aerobic conditions. The consideration of a paradigm shift, according to which lactate is the real end-product of glycolysis, independent of the presence or absence of oxygen, could bridge the great divide between those who separate glycolysis into two outcomes and those who profess that there is only one, prefix-less glycolytic pathway that always ends with the production of lactate.

## 1. Introduction

Most of our knowledge about the processes of brain energy metabolism is based on decades of research performed on a tissue of lesser complexity, the skeletal muscle. This long-standing research led to the drawing of the aerobic and anaerobic glycolytic pathways, and with the use of additional tissues, such as the liver, the tricarboxylic acid (TCA) cycle and the mitochondrial oxidative phosphorylation (OXPHOS) were also discerned. Through normal science, these energy-producing metabolic pathways have become common knowledge. Although in the mid-1920s to the early 1930s some research was performed using brain tissue, it was guided mainly by results of studies performed using skeletal muscle. Therefore, the discovery that in the presence of oxygen lactate disappears from brain tissue was considered a brain mechanism to rid itself of this useless glycolytic end-product [1,2,3,4,5,6]. Later the possibility that lactate could be used, not just removed, from the tissue in the presence of oxygen was indicated, but these investigators did not pursue that possibility further [7,8]. However, a more rigorous search of older literature revealed a study by Elliott et al. [9], where they clearly demonstrated that respiring brain slices (cortex) metabolized glucose to produce lactate, which is then oxidized to CO_2_ and H_2_O. Moreover, these investigators showed that lactate could replace glucose as the substrate of respiration in these slices. This study was published three years prior to the publication of the biochemical sequence of the glycolytic pathway, yet its obscurity is an enigma. Of the 108 citations this paper has received over the past 88 years, less than 10 were related to its finding about lactate-supported brain respiration, and several of those were self-citations. One could only surmise that most scientists in this field of research used skeletal muscle as their specimen, while only a minority used brain tissue, where their findings received much less exposure. Consequently, for over eight decades, glycolysis has been taught in schools and universities almost unchanged from its original 1940 drawing, where the aerobic version ends with pyruvate and the anaerobic one ends with lactate. By the time the elucidation of the sequence of the enzymatic reactions, the substrates, and the products of the glycolytic pathway were revealed, not much was known about the role of mitochondria in ATP production. However, the sequence of the TCA cycle was already published then [9], where pyruvate was suggested to be the entry substrate of that cycle. Embden, Meyerhof, and Parnas, who drew the glycolytic pathway, were aware of that suggestion and the role of the TCA cycle in respiration. Along with the general acceptance that without oxygen, glucose breakdown ends with lactate, the useless end-product of the process, it was reasonable to accept the suggestion that in the presence of oxygen, glycolysis ends with pyruvate. Accordingly, two molecules of ATP are invested in the breakdown of one molecule of hexose (glucose) into two triose molecules (pyruvate) and the production of four molecules of ATP. Consequently, presenting aerobic glycolysis as a pathway that ends with pyruvate aligns with the suggestion by Kreb and Johnson [10] that the glycolytic pathway supplies the substrate for the TCA cycle and respiration. To account for lactate production in the absence of oxygen, the fathers of the glycolytic pathway included the lactate dehydrogenase (LDH) reaction that converts pyruvate to lactate. Could these scientists propose lactate as the real substrate of the TCA cycle if they were aware of the study by Elliott et al. [9]? Their construction was clearly based on the old supposition that lactate is a useless byproduct. Hence, glycolysis was proposed to have two separate outcomes, an aerobic one that ends with pyruvate, and an anaerobic one that ends with lactate. This division is still being taught and recited today both in textbooks and biochemistry courses in classrooms and online. The present perspective advocates against the old doctrine of two separate glycolytic outcomes, by employing studies performed over the past four decades, validating the need for a paradigm shift. Accordingly, there is only one glycolytic pathway that always ends with lactate, independently of the presence or absence of oxygen. Moreover, this essay aims to caution against the continued devotion of many investigators and teachers to the old paradigm, a loyalty that impedes scientific progress in this utmost important field of brain research [11]. Therefore, this perspective focuses on brain glycolysis, its end-product, lactate, and its function as a principal oxidative mitochondrial substrate of the TCA cycle and OXPHOS. Nevertheless, many of the arguments raised here could be applied to other tissues and organs such as skeletal muscle, heart, and lungs. Obviously, this monograph does not cover other alternative oxidative energy substrates available for brain mitochondria. Readers can find an excellent recent review article that covers these substrates and other related topics [12]. Moreover, this perspective is not necessarily a balanced comparison between the different views and concepts that divide the research field of brain energy metabolism. Such comparisons have been covered over the past decade in many excellent review articles, some of which are cited in the following pages.

## 2. To Shift or Not to Shift a Paradigm?

Since its discovery in 1780 by Carl Wilhelm Scheele, lactate carried the negative reputation of milk spoiler, a useless product of anaerobic glycolysis, and even a poisonous substance. Then Brooks [13] published his notable study, showing that lactate is shuttled from fast twitch muscle fibers, where it is produced, to slow twitch ones, where it is utilized as an energy substrate during sustained exercise in mammals. Independently, Schurr et al. [14] demonstrated in vitro the ability of neurons to sustain their function, utilizing lactate as their sole oxidative energy substrate. This study demonstrated that lactate supports not just respiration, as shown by Elliott et al. [9], but also neuronal activity. Less than a month later, Fox et al. [15] published their seminal study, claiming that brain activity is supported by non-oxidative glucose consumption (glycolytic ATP production). These three studies, published in a span of three years, seem to shake the tranquility of the research field of energy metabolism in general and that of the brain in particular, a response that could happen 50 years earlier, if the study by Elliott et al. [9] had received the attention it deserved. Suddenly, lactate has been viewed in a completely new light, an energy substrate rather than a waste product, and the active brain, believed to require significant increases in ATP supply, had been shown to be supported by the inefficient glycolytic pathway alone. Naturally, before such revolutionary ideas could be accepted, both had faced much skepticism. While skepticism is part and parcel of scientific research, these two key findings, that lactate is an oxidative energy substrate, and that the brain does not require more than a meager amount of ATP to support its activity, have divided the scientists researching brain energy metabolism over the past four decades. While our knowledge about the many roles of lactate in the brain expanded over this period [16,17,18,19,20,21], one particular construct from the original 85-year old chart of the glycolytic pathway stubbornly remained unchanged, despite it probably being erroneous, i.e., assigning two different end-products, pyruvate or lactate, depending on the presence or absence of oxygen, respectfully, as has already been described in the Introduction section above. Embden, Meyerhof and Parnas must have also been familiar with the Pasteur Effect, the slowdown of glycolysis by oxygen, which probably made their decision to end aerobic glycolysis with pyruvate easier, although the specific mechanism by which aerobiosis inhibits glycolysis was yet to be discovered. This effect of oxygen on glycolysis does not involve the enzyme LDH, but rather the allosteric inhibition of phosphofructokinase by ATP. More than eight decades later, the original division of glycolysis into aerobic and anaerobic pathways is still cited, even in recent publications, and taught as such [22,23,24,25] despite the overwhelming evidence that neither oxygen nor mitochondria could prevent glycolysis from proceeding to end up with the production of lactate, as summarized by several review articles [26,27,28,29,30].

Brooks et al. [29] stated this paradox as follows:

“*Contemporary textbooks of biochemistry and physiology abound the 19th-century concepts that metabolism is either “anaerobic” (without O_2_) or “aerobic” (with O_2_). Glycolytic flux from glucose and glycogen is typically depicted in textbooks as progressing to pyruvate and then to the tricarboxylic acid (TCA) cycle. However, if oxygen is absent, textbooks assert that glycolysis progresses to lactate. This is a convenient motif, typically copied by one textbook author from another and then through serial editions of texts. Amazingly, some textbook authors who advanced the idea of lactate production due to oxygen limitation were biochemists who worked with cells in high-glucose-containing culture media under fully aerobic conditions of one atmosphere pressure in which the partial pressure of oxygen was at least 50% greater than in the arterial blood of individuals at sea-level altitudes. As the maintenance of cells in such preparations required the daily changing of the incubation media to maintain high [glucose], low [lactate], and physiological pH, the observations should have informed the investigators that lactate was produced under fully aerobic conditions. Contemporaneously, physiologists measured lactate [L] to pyruvate [P] concentration ratios (L/P) of 10 in muscles and blood in resting mammals, including humans, and further observed the L/P to rise more than 100 during submaximal exercise. However, did any of the textbook authors ever look to determine whether isolated mitochondria oxidize lactate? For many textbook authors, the answer is “no.” However, for a few others, the answer is “yes”. Moreover, some investigators also looked for the presence of a mitochondrial monocarboxylate (lactate) transporter (mMCT) and a lactate dehydrogenase (mLDH) enzyme. Unfortunately, while some attempts failed, fortunately, other attempts to observe mitochondrial lactate oxidation and the presence of mLDH and mMCT were successful. Importantly, lactate oxidation in human muscle mitochondrial preparations was observed. Regrettably, for a time, positive results were overlooked or castigated as being “controversial” and dismissed for failing to fit with established paradigms of metabolic regulation*.”

Passarella [31] expresses a similar sentiment in his recent review:

“…*the existence of mitochondrial L-lactate metabolism, mediated by specific L-lactate transporters and a mLDH, remains largely overlooked in contemporary biochemistry textbooks and in several recent reviews and research papers*.”

A significant number of scientists in the field of energy metabolism persistently hold to the concept that oxygen could prevent the conversion of pyruvate to lactate, a concept on which a great deal of “common knowledge” relies, too. When wrong common knowledge becomes entrenched in the minds of the investigators who use it in their studies and their teachings, in the minds of their students, and therefore in the minds of the public, rectifying it could be an overwhelming task. Consequently, “aerobic glycolysis,” as described in many sources today, could end either with pyruvate, or with lactate, where the latter signifies a non-oxidative glucose consumption [15], an outcome that in the past was believed to be a trait of cancerous cells only [32,33]. This convoluted situation could be rectified easily if all agreed that glycolysis, under any condition, begins with the phosphorylation of D-glucose by the enzyme hexokinase, and ends with the reduction of pyruvate to L-lactate by LDH, a process carried out by an enzymatic conglomerate of eleven enzymes located in the cell cytosol. Either prefix, aerobic or anaerobic, are not necessary to describe the pathway that, thermodynamically (see below), should end with lactate [34,35]. Oxygen, even at the highest possible concentration, cannot block the LDH reaction, as is clear from the glycolysis of red blood cells or the activity of the Na^+^/K^+^-ATPase pump that is supported by its own ATP-producing glycolytic pathway, where lactate in both systems is the end-product. Nevertheless, as illustrated below by some relatively recent studies, the concept of aerobic glycolysis as a pathway that ends with pyruvate is still deeply rooted. Table 1 lists several recent publications that exemplify the continued reliance of many in the research field of brain energy metabolism on the original 1940 paradigm of glycolysis, according to which under aerobic conditions the pathway starts with the breakdown of a molecule of glucose and ends with two molecules of pyruvate.

The fact that many within this field continue to argue for two separate glycolytic outcomes, while accepting the fact that even under conditions where the pathway is supposed to end with pyruvate, it ends with lactate (aerobic glycolysis), requires a re-evaluation of the accepted dogma. ‘Habit of mind’ [42] has been discussed before as a possible explanation for the resistance to shift away from said doctrine when scientific findings justify a paradigm shift [27,28,29,31,43]. Clearly, neither the thermodynamic argument nor a psychological explanation could overcome this resistance for said shift. Therefore, it is up to a scientific experiment to show which glycolysis is the real pathway: (a) the one composed of eleven sequential enzymatic reactions that begins with the phosphorylation of one equivalent of glucose and ends with the formation of two equivalents each of lactate, ATP and NAD^+^ or: (b) the glycolytic pathway that ends after its tenth enzymatic step, the conversion of phosphoenolpyruvate (PEP) to pyruvate when both oxygen and mitochondria are present. While there are ample experiments to support the former, there are a lack of experiments to support the latter. The following sections of this essay highlight why the research field of brain energy metabolism would thrust forward when its practitioners accept glycolysis as a pathway that begins with glucose and ends with lactate.

## 3. Paradigm Shift: Glycolysis Always Ends with Lactate Regardless of Oxygen Presence or Absence

**Figure 1:**
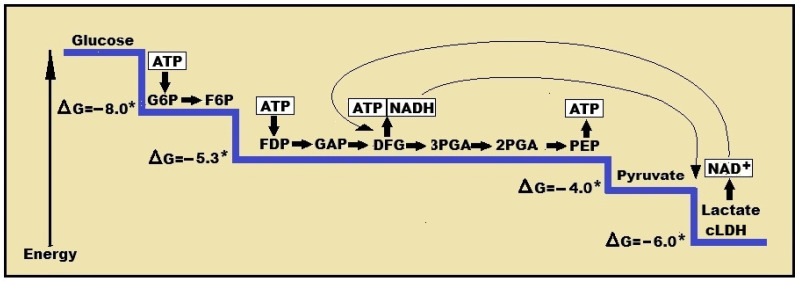
A schematic presentation of the glycolytic free energy change through the eleven enzymatic steps of the pathway. The first step of glycolysis, the phosphorylation of glucose to glucose-6-phosphate by hexokinase, produces the largest free energy change (ΔG = −8.0 Kcal/mol). The last step, the conversion of pyruvate to lactate by lactate dehydrogenase (LDH) produces the second largest free energy change (ΔG = −6.0 Kcal/mol). The conversion of pyruvate to lactate also produces NAD^+^, keeping up with the cyclical nature of the pathway, while supplying the redox cofactor for additional redox reactions. NAD^+^ = nicotinamide adenine dinucleotide; NADH = nicotinamide adenine dinucleotide, reduced form; cLDH = cytosolic lactate dehydrogenase. ATP = adenosine triphosphate; G6P = glucose 6-phosphate; F6P = fructose 6-phosphate; FDP = fructose 1,6-biphosphate; GAP = glyceraldehyde 3-phosphate; DFG = 1,3-biphosphoglycerate; 3PGA = 3-phosphoglycerate; 2PGA = 2-phosphoglycerate; PEP—phosphoenolpyruvate; * non-equilibrium reaction; ΔG = free energy change in Kcal/mol.

Thermodynamically, glycolysis must flow through all its enzymatic reactions, including the very last one, the LDH reaction (Figure 1). Park et al. [34] investigated several metabolic pathways in three different organisms, including mammals. They concluded that all metabolic pathways, their metabolite concentrations, fluxes and free energies, imply an efficient enzyme usage, which achieved by maintaining a negative Δ*G* throughout. While the investigators did not specifically measure or discuss the last enzymatic reaction of glycolysis, i.e., the conversion of pyruvate to lactate by the cytosolic LDH (cLDH), the significant free energy gained by this reaction going forward (Δ*G* = −6.0 Kcal/mol) aligned with their general conclusion i.e., the glycolytic pathway should end with lactate. Nevertheless, upon examining Figure 6D of that study [38], two questions arise: 1. Why does glycolysis in this figure end with pyruvate prior to its conversion to acetyl-CoA and its entry into the TCA cycle, while lactate is shown as a side end-product that goes nowhere? 2. Could the investigators, using their tools and approaches, decipher whether the glycolytic process proceeds from glucose to pyruvate to acetyl-CoA or it proceeds from glucose to lactate and then back to pyruvate (intra-mitochondrially) and acetyl-CoA? One measure that clearly indicates glycolysis tendency to proceed through eleven enzymatic reactions ending with lactate production is the ratio lactate/pyruvate in the brain and elsewhere. Although this ratio fluctuates, values of cerebrospinal fluid (CSF) lactate concentration are always significantly higher than pyruvate. For instance, lactate to pyruvate ratio measured in 627 patients over ten years, fluctuated from 9.05 to 26.37 [44]. Another study of 197 children found this ratio to be 16.9 to 19.2 [45]. Similar ratios were recorded in other tissues such as the skeletal muscle in rest, and much higher during exercise [29]. These values signal that under normal circumstances lactate is produced at levels 10 times or higher than pyruvate, signaling that the former is the main glycolytic product, and therefore, in agreement with the thermodynamic argument.

In a personal communication with one of the authors of the above-cited study [34], the answers to these two questions were: “At the whole-body level, we see that a majority of the glycolytic process goes straight from glucose to pyruvate to acetyl-CoA in the fed state (some also to lactate), whereas in the fasted state it mainly proceeds to lactate (which enters the bloodstream) and then back to pyruvate (presumably extra-mitochondrially) and to acetyl-CoA”. These answers do not align with the principle of their own study according to which efficient enzyme usage of a metabolic pathway is achieved by maintaining a negative Δ*G* throughout. Claiming that glycolysis stops at pyruvate rather than proceeds to lactate contradicts this principle. In addition, the presumption, held by many, that the conversion of lactate back to pyruvate occurs extra-mitochondrially, is wrong, since that enzymatic reaction in the cytosol could not be catalyzed by cLDH against its negative Δ*G*. In contrast, intra-mitochondrially, lactate conversion to pyruvate by mitochondrial LDH (mLDH) does proceed fluently [29,46,47,48,49,50,51,52,53]. A recent study demonstrated that lactate also plays a role in suppressing glycolysis, while activating the mitochondrial electron transport chain independently of its metabolism [54].

Accepting glycolysis as a pathway that always ends up with lactate means that its prefixes, aerobic and anaerobic, are meaningless [28,30]. Accordingly, as has been mentioned earlier, under oxidative conditions, lactate is the initial substrate for the mitochondrial TCA cycle and OXPHOS. Moreover, when ample supply of lactate is available to the brain, it becomes the preferred substrate for oxidative energy production over glucose [55,56,57,58,59,60,61,62]. Only when lactate is the final glycolytic product that the pathway fulfills its cyclic purpose of renewing NAD^+^ supply [52]. The conversion of lactate back to pyruvate, and then to acetyl-CoA, occurs in the mitochondrion upon lactate transport via a monocarboxylate transporter (MCT) into the organelle and its oxidation by mLDH, a reaction that is not part of the glycolytic pathway.

Yet old habits are still at the center of the debate in the field of brain energy metabolism, where it has always been easy to accept the fact that with oxygen the glycolytic pathway ends with pyruvate, while without oxygen it ends with lactate [29]. And since without oxygen, mitochondrial activity is impossible, the only metabolic pathway capable of producing ATP is glycolysis. However, the Warburg effect [32,33] describes aerobic glucose consumption in cancerous cells, where lactate is the main product, the process by which these cells produce most of the energy required for their proliferation. This phenomenon was discovered more than a decade before the glycolytic pathway sequence was elucidated, but the scientists of the day studying energy metabolism consider it unique to cancerous cells. However, nowadays ‘aerobic glycolysis’ is a term used to describe glycolytic glucose consumption that ends with the production of lactate by normal, noncancerous cells, that are fully oxygenated. Despite the term’s popularity, it is a metabolic process that defies decades of accepted concept, according to which ‘aerobic glycolysis’ is supposed to end with pyruvate, as every biochemistry textbook describes, and similarly in many scientific articles. While oxygen alone cannot stop glycolysis at the production of pyruvate (see glycolysis of red blood cells), when mitochondria are present textbooks state that pyruvate is the glycolytic end-product. Notwithstanding, aerobic glycolysis in today’s jargon means a process that ends with lactate despite the presence of both oxygen and mitochondria, as was first described by Fox et al. [15]. This description was based on an indirect estimate of oxygen consumption, calculated from the measurement of changes in brain blood oxygen level and/or the rate of cerebral blood flow (CBF). Regardless, a significant number of investigators have accepted aerobic glycolysis as the metabolic process that provides the additional ATP necessary for brain activity, and to rationalize the brain’s choice of an inefficient ATP-producing process for that task, the efficiency tradeoff hypothesis was offered [63]. The arguments for and against the validity of the ‘aerobic glycolysis’ concept have been recently reviewed [28]. Additionally, the results of two recent studies [64,65] demonstrate that myelin can store enough oxygen, which is available for the active brain. These studies support previous findings [55] that oxygen is consumed upon brain activation, possibly explaining the non-reliance of the active brain on an immediate availability of blood-borne oxygen [66]. In essence, these studies challenge not only the efficiency tradeoff hypothesis, but they also highlight the inadequacy of techniques such as BOLD fMRI and CBF measurements to gauge immediate brain oxygen consumption accurately, since these techniques cannot sense and measure consumption of oxygen stored in brain tissue outside the vasculature.

## 4. The Roles of MCT and mLDH in Mitochondrial Lactate Metabolism

The existence of mLDH in rat heart, kidney, liver, and lymphocytes mitochondrial preparations was first reported over three decades ago [67]. However, the study claimed that the presence of mLDH in brain mitochondrial preparation was inconclusive. Our knowledge of mLDH in muscle, heart, and liver tissues was expanded when a detailed study showed that there are differences in the isoform composition of LDH, not only between cLDH and mLDH, but also between liver, heart and muscle mLDH [46]. These findings were confirmed a year later, suggesting that the direction of LDH reaction, reduction in pyruvate to lactate, or oxidation of lactate to pyruvate, is determined not just by LDH isoform composition, but also by its compartmentalization and its association with specific cellular structures [68]. An in vitro study, employing acute rat hippocampal slices and LDH inhibitors [47] strongly suggested that mLDH, not cLDH, participates in the- oxidation of lactate to pyruvate that enters the TCA cycle after its conversion to acetyl CoA. Later, other studies demonstrated the existence of brain mLDH [48,69]. A review article summarized the mitochondrial lactate dehydrogenase research affair in detail [49]. For lactate to be oxidized intra-mitochondrially, it must be transported into the organelle first. A description of the distribution, function, and regulation of the specific monocarboxylate transporters (MCTs) of the central nervous system was published [70]. A recent study describes with greater detail the distribution and function of the four brain MCTs, belonging to the SLC16 family of solute carriers, that catalyzes proton-coupled lactate transport [71]. Accordingly, MCT1 is expressed almost ubiquitously in the brain, in endothelial cells, astrocytes, and oligodendrocytes. Astrocytes release lactate via MCT4 and possibly into oligodendrocytes via MCT1. MCT2 catalyzes the proton-coupled transport of monocarboxylates such as lactate, pyruvate, and ketone bodies, across the plasma membrane, showing the highest affinity for lactate followed by pyruvate. MCT2 is expressed mainly in neurons but also found in astrocytes. It has been shown that MCT1 is strongly expressed in astrocytes of mice, while MCT2 is expressed in neurons, findings that are consistent with the ANLS hypothesis, where lactate is transported out of astrocytes via MCT1 and is taken up by neurons via MCT2 [72]. MCT4 is a low-affinity, high-capacity transporter and is expressed mainly in astrocytes [73] and is known to facilitate lactate excretion in cells that have high rates of glycolysis [74]. MCT2 is known to be a transporter with a higher affinity for most monocarboxylates than MCT1 [75]. In short, the distribution of MCTs in the plasma membrane of neurons and astrocytes suggests a significant role of these transporters in the shuttling of energy metabolites between these two cell types. A study that used confocal laser scanning microscopy and immunoblotting after immunoprecipitation from cell lysates demonstrated that the location of MCT1, MCT2 and LDH is in neuronal mitochondria, and additionally that these MCTs and LDH are associated with cytochrome oxidase (COX) in rat brain mitochondria [48]. As mentioned above LDH exists in several isomers that differ from one another based on the type of the four subunits that compose them. This subunit composition also determines the direction of the reaction they catalyze, pyruvate to lactate or vice versa. There are several genes that code for the synthesis of the LDH protein, two are found in most tissues and organs, including the brain, *LDHA* and *LDHB* [76]. The former’s product is LDHA, also known as LDH-M (M for muscle), and the latter is LDHB, also known as LDH-H (H for heart). Accordingly, five different isomers could be formed, each composed of four subunits of LDH; LDH-1, composed of four M subunits, LDH-2, composed of three M subunits and one H subunit, LDH-3, composed of two M and two H subunits, LDH-4, composed of three H subunits and one M subunit, and LDH-5 composed of four H subunits. LDH-M has higher affinity to pyruvate and therefore catalyzes its conversion to lactate, with an oxidation of NADH to NAD^+^, while LDH-H has higher affinity to lactate and its conversion to pyruvate with a reduction in NAD^+^ to NADH. Studies showed the colocalization of MCTs and LDH isoforms in the inner mitochondrial membrane of rat-derived L6 skeletal muscle cell line [77] and in mitochondria from untransformed primary breast cell line, HMEC 184 [78]. Nevertheless, it is now recognized that mitochondrial LDH (mLDH) is a constituent of the mitochondrial proteome [79]. A study using ultrasensitive sensors showed that lactate is highly enriched in mitochondria at levels that significantly exceed those in the cytosol [80]. Clearly, there is overwhelming evidence demonstrating the existence of a complete mitochondrial machinery that imports and metabolizes lactate, which cannot be ignored or discounted any longer.

**Figure 2:**
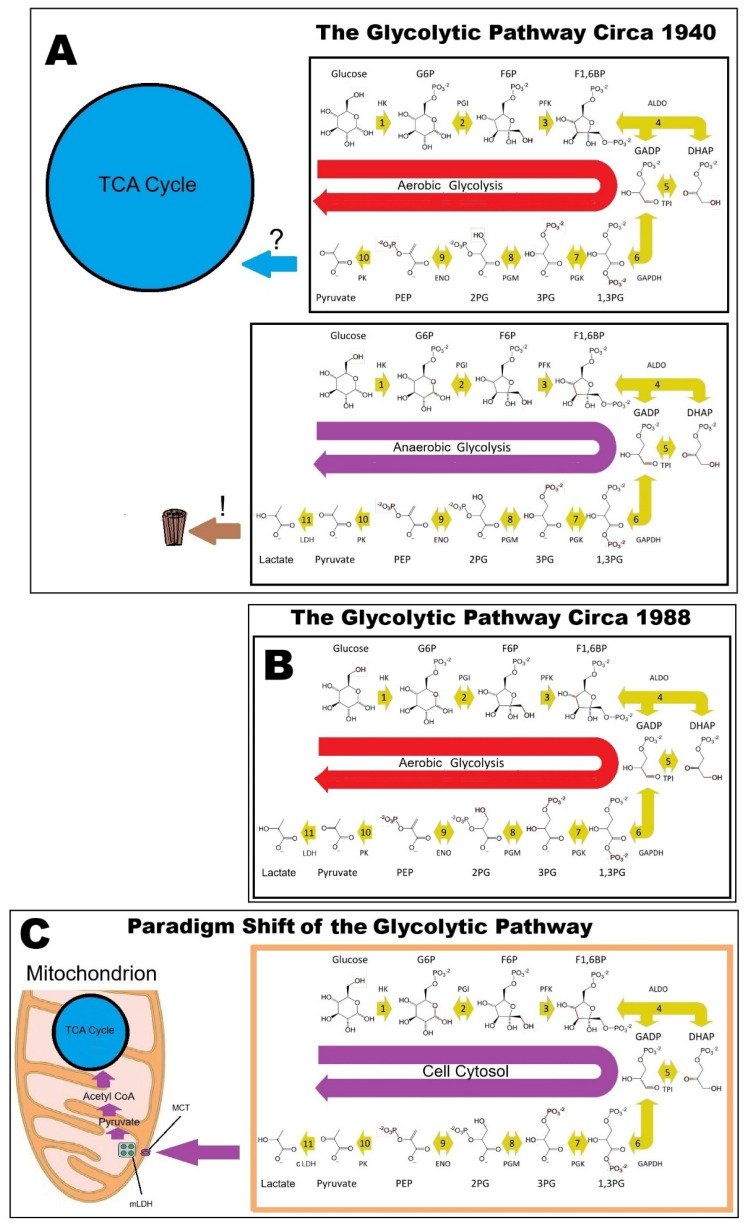
A schematic presentation of the historical progress of our knowledge and understanding of glycolysis. (**A**) The original presentation of the glycolytic pathway sequence by Embden, Meyerhof and Parnas in 1940, where two separate outcomes were offered, an aerobic one that ends with the production of pyruvate, and an anaerobic one, which ends with the production of lactate. The decision to place pyruvate as the end-product of aerobic glycolysis was made based on the suggestion by Krebs and Johnson [9] that pyruvate is the substrate for the TCA cycle. Lactate at the time was deemed to be a waste product! (**B**) In 1988, Fox et al. [15] published their discovery according to which the active brain uses non-oxidative glucose consumption to fuel its increased energy needs, ushering in the term ‘aerobic glycolysis’ to mean glycolysis that ends with lactate despite the presence of oxygen, adding unnecessary confusion to the terminology already in use when glycolysis is studied and taught. (**C**) Research over the past four decades has compelled investigators to realize the need for a paradigm shift in the glycolytic pathway’s presentation to accommodate the new discoveries. The pathway sequence is copied from Wikipedia with several modifications. TCA cycle = tricarboxylic acid cycle; HK = hexokinase; G6P = glucose 6-phosphate; PGI = phosphoglucose isomerase; F6P = fructose 6-phosphate; PFK = phosphofructokinase; F1,6BP = fructose 1,6-biphosphate; ALDO = aldolase; DHAP = dihydroxyacetone phosphate; GADP = glyceraldehyde 3-phosphate; TPI = triose phosphate isomerase; 1,3-PG = 1,3-biphosphoglycerate; PGK = phosphoglycerate kinase; 3GP = 3-phosphoglycerate; PGM = phosphoglycerate mutase; 2PG = 2-phosphoglycerate; ENO = enolase; PEP = phosphoenolpyruvate; PK = pyruvate kinase; cLDH = cytosolic lactate dehydrogenase; mLDH = mitochondrial lactate dehydrogenase; MCT = monocarboxylate transporter.

Figure 2 illustrates the historical development of our knowledge and understanding of glycolysis from the elucidation of its sequence to the paradigm shift that is supported by multiple studies published over the past four decades.

## 5. Conclusions

The past four decades have brought about a paradigm shift in the field of brain energy metabolism research. Unfortunately, a significant number of investigators within this field continue to cling to an 85-year-old paradigm, relying on it for the interpretation of their studies. Consequently, a long-standing debate between those who accept the new paradigm and those who reject it continues to divide investigators of this utmost important field of research. This endeavor set forth to understand how the brain, the one organ that consumes more energy than any other, controls its energy needs in health, during rest and activity, and how the loss of this control leads to numerous brain disorders. At the center of the debate stands the first metabolic pathway to be fully explicated in 1940, namely, glycolysis. All enzymes, cofactors, substrates and products were identified and arranged in the order they comprise the pathway. Although it was clear then that, anaerobically, this pathway ends with the conversion of pyruvate to lactate, it was assumed that the presence of oxygen should make this last step unnecessary. The idea of two different glycolytic outcomes had become deeply rooted in textbooks, school and college classes, and in the minds of both experts and novices. Despite the continuous accumulation of evidence in support of a required paradigm shift as described above, the research community of brain energy metabolism remained divided. A recent study in humans [81] concluded that “…when lactate is made available in circulation—whether through passive infusion or exercise—the healthy human brain preferentially metabolizes it over glucose to sustain energetic needs. This sparing effect of lactate on glucose appears to permit glucose to fulfil non-energetic requirements of the brain.” It is too early to determine if findings such as this would sway more investigators to accept the new paradigm. The publication of a recent editorial attempted to bridge this divide [11]. Considering the responses that followed shortly after its publication [82,83], it appears that additional efforts to build a bridge are needed. At least two principal issues must be clarified experimentally by the investigators who support the non-oxidative glucose consumption during brain activity. First, a direct measurement of glucose consumption combined with the direct measurement of oxygen consumption or CO_2_ production of the active brain should be performed. The results would either support or reject the claim that the active brain relies on a meager amount of ATP to fuel its increased energy needs. Indirect measurements of oxygen consumption, using BOLD fMRI or CBF, are not satisfactory as they do not detect any consumption of non-vascular oxygen. Second, a direct measurement of pyruvate production of the resting brain accompanied by relatively small production of lactate if any. Alternatively, an experimental demonstration showed that in the resting brain under fully oxygenated conditions, the activity of the glycolytic LDH is inhibited. If the active brain consumes glucose and oxygen concomitantly, then the claim for a non-oxidative glucose consumption by the active brain is wrong. If the glycolytic pathway of the resting brain does not end with the production of pyruvate or if oxygen does not inhibit the activity of the glycolytic LDH, then the concept of two separate outcomes of glycolysis, aerobic and anaerobic, should be abolished from biochemistry textbooks and classes.

This opinionated perspective is another attempt to correct this flawed concept, which hinders the progress and understanding of the utmost important processes that energize the brain both in rest and during activity, in health and disease. It weighs the discoveries that made us question the old archetype and details the scientific evidence that justifies the following paradigm shift: Glycolysis is a metabolic pathway of eleven enzymatic reactions that begins with glucose and ends with lactate. Neither oxygen nor mitochondria, whether present or absent, affect its outcome.

## Figures and Tables

**Table 1:** Six recent publications, four of which are highly cited, where the original depiction of aerobic glycolysis by Embden, Meyerhof and Parnas, is still promoted.

1. A study titled “Metabolic Characterization of Acutely Isolated Hippocampal and Cerebral Cortical Slices Using [U−^13^C]Glucose and [1,2−^13^C]Acetate as Substrates,” [36] found a high production of lactate by both hippocampal and cortical slices that reached its maximum after 15 min of incubation with [U−^13^C]Glucose, a production that did not increase throughout the following 75 min.* While they correctly pointed out that lactate production is due to glycolytic activity, they failed to explain why lactate production, as measured by its content in the incubation medium, did not continue to increase above the initial level measured at 15 min, which indicates lactate utilization by both hippocampal and cerebral cortical slices. Clearly, the investigators never considered the utilization of lactate as the mitochondrial substrate to explain this observation. (Cited 43 times since its publication). * Interestingly, the findings of McNair et al. [36] are very similar to those of Elliott et al. [9], although the latter explained the lack of continuing rise in lactate concentration as oxidative lactate utilization.
2. In an article titled “Fueling thought: Management of glycolysis and oxidative phosphorylation in neuronal metabolism” [37], the author defined glycolysis as the partial metabolism of glucose to pyruvate or lactate that occurs in the cytosol (and that possibly is sub-compartmented within the cytosol, perhaps to the intracellular surface of the plasma membrane). He detailed that metabolism of glucose to pyruvate yields two net phosphorylation of ADP to ATP and two 2*e*^−^ (two-electron), reductions in NAD^+^ to NADH, and that sustaining glycolysis requires reoxidation of NADH to NAD^+^ either through the coordinated reduction in pyruvate to lactate by lactate dehydrogenase (LDH) or through the action of one of the mitochondrial NADH shuttles. (Cited 411 times since its publication).
3. In a study titled “Metabolomics and isotope tracing” [38], the investigators used the isotope [1,2−^13^C]glucose to trace glycolytic products. While tracing lactate, they relied on the concept that pyruvate is the glycolytic end-product that enters the TCA cycle, as illustrated in Figure 6D of this article [38]. In a personal communication, Prof. Rabinowitz stated: “At the whole-body level, we see that a majority of the glycolytic process goes straight from glucose to pyruvate to Acetyl-CoA in the fed state (some also to lactate), whereas in the fasted state it mainly proceeds to lactate (which enters the bloodstream) and then back to pyruvate (presumably extra-mitochondrially) and to Acetyl-CoA.” However, the study’s results could very easily be explained by accepting the concept that glycolysis ends with lactate, the monocarboxylate that is transported by MCT into the mitochondria, where it is readily converted back to pyruvate via the mitochondrial LDH (mLDH), which then enters the TCA cycle via acetyl-CoA. (Cited 941 times since its publication).
4. A detailed review article summarized the progress in research of “Retinal energy metabolism in health and glaucoma” [39]. It graphically illustrates glycolysis as a pathway that ends with pyruvate, while simultaneously the authors accept the idea that retinal lactate produced glycolytically is utilized as the mitochondrial oxidative substrate, a somewhat contradicting statement. (Cited 125 times since its publication).
5. A detailed study titled “Divergent Cellular Energetics, Glutamate Metabolism, and Mitochondrial Function Between Human and Mouse Cerebral Cortex” [40], presents glycolysis graphically as a pathway that begins with the phosphorylation of glucose and ends with the production of pyruvate, although the investigators also used β-hydroxybutyrate (BHB) as a mitochondrial oxidative substrate, which is not different from the ability of their mitochondrial preparation to use lactate. Unfortunately, the investigators did not consider lactate as a potential mitochondrial substrate. (Cited 20 times since its publication).
6. An encompassing review titled “Brain energy metabolism: A roadmap for future research” [41], emphatically promotes the concept of glycolysis with pyruvate as its final product and the mitochondrial substrate of the TCA cycle. Among the 26 co-authors of this review are many established investigators in the research field of brain energy metabolism. Their review minimizes the role of lactate as a significant energy substrate and question the validity of the astrocyte neuron lactate shuttle hypothesis. (Cited 80 times since its publication)

## Data Availability

No new data were created or analyzed in this study. Data sharing is not applicable to this article.
